# The risk of preterm birth in combinations of socioeconomic position and mental health conditions in different age groups: a Danish nationwide register-based cohort study

**DOI:** 10.1186/s12884-021-04138-0

**Published:** 2021-10-14

**Authors:** Camilla Klinge Knudsen, Amanda Marie Somer Christesen, Signe Heuckendorff, Kirsten Fonager, Martin Nygård Johansen, Charlotte Overgaard

**Affiliations:** 1 Department of Social Medicine, Aalborg University Hospital, Havrevangen 1, 9000 Aalborg, Denmark; 2 Public Health and Epidemiology Group, Department of Health Science and Technology, Aalborg University, Aalborg, Denmark; 3 Department of Clinical Medicine, Danish Center for Clinical Health Services Research (DACS), Aalborg University, Aalborg, Denmark; 4 Department of Clinical Medicine, Aalborg University, Aalborg, Denmark; 5 Unit of Clinical Biostatistics, Aalborg University Hospital, Aalborg, Denmark

**Keywords:** Preterm birth, Mental health conditions, Maternal mental health, Socioeconomic position, Educational level, Maternal age, Inequality, Birth outcome, Pregnancy, Additive interaction

## Abstract

**Background:**

Inequality in preterm birth is a world-wide challenge that has proved difficult for maternity care services to meet. Reducing the inequality requires identification of pregnant women at particularly high risk of preterm birth in order to target interventions. Therefore, the aim was to estimate the risk of preterm birth in women with different combinations of socioeconomic position, mental health conditions, and age.

**Methods:**

In this nationwide register-based cohort study, we included all first-time mothers that gave birth to a singleton liveborn infant in Denmark between 2000 and 2016. The absolute and relative risk of preterm birth (< 37 weeks of gestation) was examined in different combinations of educational level (high, intermediate, and low) and mental health conditions (no, minor, and moderate/severe) in three age strata (≤23, 24–30, and ≥ 31 years). We estimated the relative risk using Poisson regression with a robust error variance. As additive interaction can help identify subgroups where limited resources can be of best use, we measured the attributable proportion to assess the risk that is due to interaction of the different exposures.

**Results:**

Of the 415,523 included first-time mothers, 6.3% gave birth prematurely. The risk of preterm birth increased with decreasing educational level and increasing severity of mental health conditions in all age strata, but most in women aged ≥31 years. The highest absolute risk was 12.9% [95% CI: 11.2;14.8%] in women aged ≥31 years with low education and moderate/severe mental health conditions resulting in a relative risk of 2.23 [95% CI: 1.93–2.58] compared to the unexposed reference group in that age strata. We found positive additive interaction between low education and mental health conditions in women aged 24–30 and ≥ 31 years and between age ≥ 31 years and combinations of mental health conditions and educational levels.

**Conclusion:**

The inequality in preterm birth increased with increasing age. To reduce inequality in preterm birth focused attention on women with higher age further combined with lower educational levels and mental health conditions is essential.

**Supplementary Information:**

The online version contains supplementary material available at 10.1186/s12884-021-04138-0.

## Introduction

In developed countries, overall, 8.6% of all livebirths are born preterm [1], however, inequality is pervasive [2, 3]. As preterm birth is associated with higher mortality [4], poorer neurological development, behavioural, social, and learning difficulties [5], being born preterm presents a threat to children’s health and ability to reach their life potential. A socioeconomic gradient in the risk of preterm birth is well documented [2] even in countries with universal access to antenatal care [6, 7]. Inequity in preterm birth thus presents a significant public health challenge requiring identification of pregnant women at particularly high risk of preterm birth in order to target interventions.

When examining inequality in preterm birth, epidemiological studies have generally considered single risk factors [8] without considering that these might interact. In this way, disadvantaged socioeconomic position, mental health conditions, and younger and older age are all found to be independent risk factors of preterm birth [2, 9, 10]. In pregnant women, disadvantaged socioeconomic position is associated with both young maternal age and mental health conditions [11]. Generally, mental health conditions are the leading cause of illness among women aged 15 to 44 years [12]. In developed countries, 15.6% of all pregnant women experience a mental health condition [13], but young pregnant women are at particularly high risk [14]. Despite these associations between socioeconomic position, mental health conditions, and age, it is unknown whether these independent risk factors interact in their contribution to the inequality in the risk of preterm birth. Analyses of additive interaction can clarify this by examining whether the observed joint effects of the exposures are greater or less than the expected based on summing their independent effects on preterm birth [15]. In this way, examining additive interaction can help identify subgroups where limited resources can be of best use [16]. Therefore, it is a highly relevant public health measure [17] although it is not commonly used.

To identify relevant subgroups for intervention in order to reduce inequality in preterm birth, the aim of this study was to estimate the risk of preterm birth according to different combinations of socioeconomic position and mental health conditions in different age groups and examine additive interaction between these three risk factors.

## Methods

### Design

This study was a Danish nationwide register-based cohort study.

### Setting

In the Danish tax-based healthcare system [18], the antenatal care is free of charge and used by the majority of all pregnant women [19, 20].

### Data sources

Data on the mother and the child was retrieved from the following nationwide registers: The Danish Medical Birth Register [21], Danish National Patient Registry [22], The Danish national prescription registry [23], Danish National Health Service Register [24], and Statistics Denmark’s registers on population and education [25]. Linkage between databases was conducted on an individual level by means of the personal registration number, a unique identifier assigned all Danish individuals and used in all public registers in Denmark enabling linkage between them [26]. Statistics Denmark conducted the linkage and anonymised data. Information on databases associated with codes and algorithms identifying exposures and outcome are available in Supplementary tables s1-s3 [Additional file 1].

### Study population

We identified all liveborn infants in Denmark in the period 1 January 2000 to 31 December 2016 and their mothers. We included first-time mothers who gave birth to a singleton liveborn infant.

**Fig. 1:**
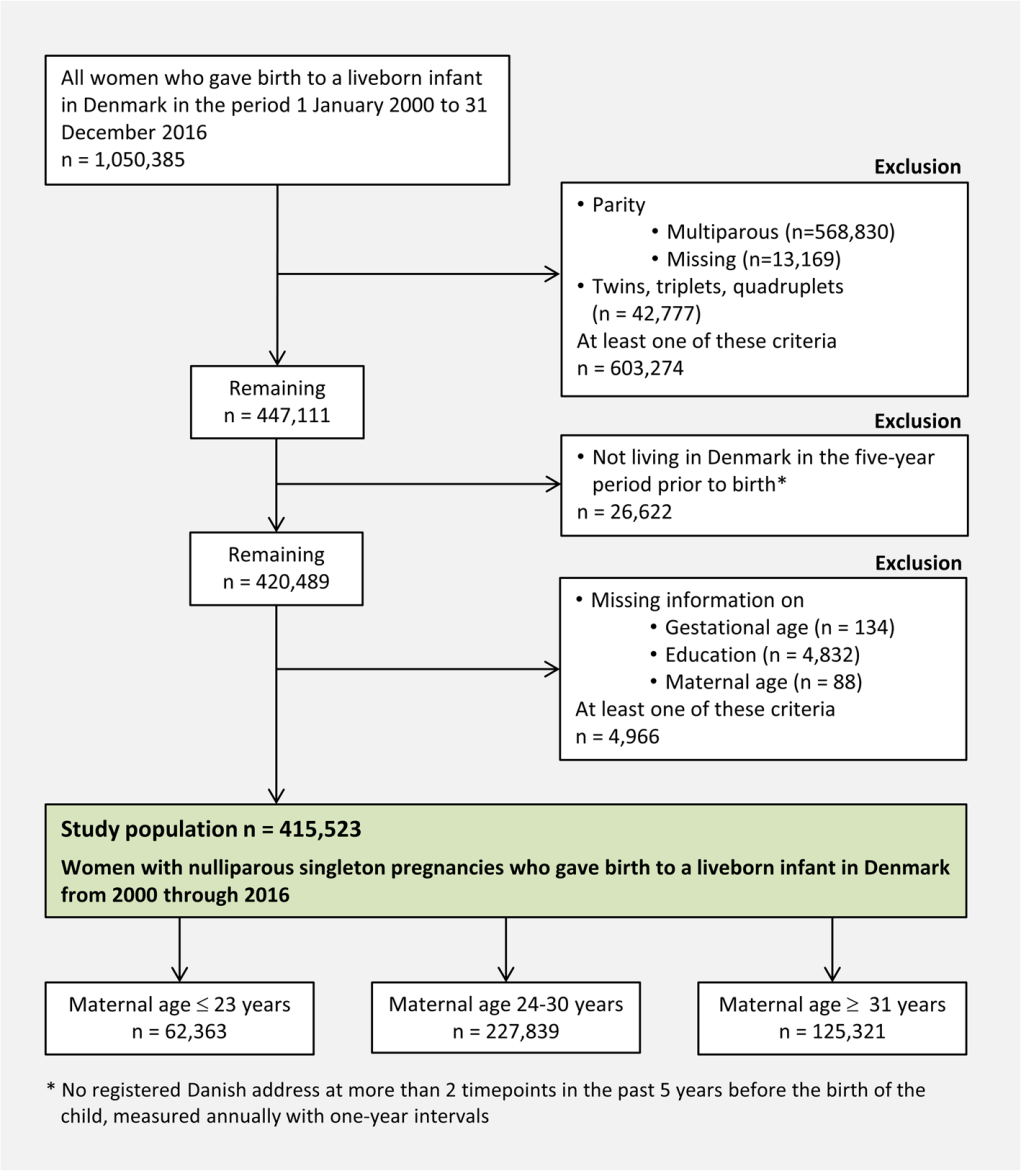
Flowchart of the inclusion, exclusion, and final study population

To increase the probability that all mental health conditions of the study population were registered, we excluded women not living in Denmark in the five-year period prior to birth as this was the period where mental health conditions were considered. Women with missing data on parity, gestational age, education, or maternal age were excluded (Fig. 1).

### Variables

The outcome measure was preterm birth, defined by the World Health Organisation (WHO) as birth before 37 completed weeks of gestation (< 259 days) [27]. In Denmark, gestational age is corrected according to early ultrasound examination [28] received by > 90% of all pregnant women [20].

Maternal age was categorised into the following three categories: ≤23, 24–30, and ≥ 31 years because of the lowest risk of preterm birth in Danish women aged 24–30 years [10].

The socioeconomic measure of interest was highest maternal educational level attained at birth of the child, as educational level is a strong predictor of preterm birth [6]. In accordance with the International Standard Classification of Education (ISCED) [29], we categorised maternal educational level into three categories: Low educational level was defined as primary school, equivalent to 10 years of mandatory education, corresponding to the ISCED level 0–2. Intermediate educational level was defined as ISCED level 3–4, consistent with secondary (high school) or vocational education. High educational level was defined as ISCED level 5–8, corresponding to a short-cycle tertiary education or above. As mean age at commencement of study at ISCED level 5 is in the mid-twenties in Denmark [30], we expected only few first-time mothers ≤23 years at the high educational level, and therefore high and intermediate education were merged for this age group resulting in two educational categories.

Mental health conditions were categorised in three mutually exclusive severity groups: Minor mental health conditions were defined as mental health conditions managed in the primary healthcare system measured as contact to private psychologist, at least two psychometric tests or two sessions of talk therapy with general practitioner, or at least two redeemed prescriptions of benzodiazepines or antidepressants. Moderate/severe mental health conditions were defined as contact to a private psychiatrist or mental health conditions managed in a psychiatric hospital (all F codes from International Classification of Diseases 10 registered as either primary or secondary diagnosis). No mental health conditions were assigned if none of the criteria above was met. Because mental health conditions often are enduring or recurrent [31], all contacts, conditions, and medication (see Supplementary table s3 in Additional file 1 for specific codes) were considered in a window of 5 years before the birth of the child.

### Statistics

Poisson regression with a robust error variance [32], was used to estimate the relative risk (RR) of preterm birth in the different combinations of education and mental health conditions. The analysis was stratified by age-group, and in all age strata the reference was women with the combination of high education and no mental health conditions. With the aim of identifying women at particularly high risk of preterm birth as relevant subgroups for intervention in order to reduce inequality in preterm birth, no adjustments were conducted as adjustment may remove important effects between social positions [33].

In order to measure additive interaction, we performed two interaction analyses where we calculated attributable proportions (AP) defined as the proportion of the risk that is due to interaction in the doubly exposed groups [16]. In the first analysis, we examined additive interaction between education and mental health conditions in each age stratum. In this analysis the doubly exposed groups (E + M+) were those exposed to low or intermediate education (E+) and minor or moderate/severe mental health conditions (M+):

$$AP=\frac{R{R}_{E+M+}-R{R}_{E+M-}-R{R}_{E-M+}+1}{R{R}_{E+M+}}=\frac{p_{E+M+}-{p}_{E+M-}-{p}_{E-M+}+{p}_{E-M-}}{p_{E+M+}}$$

p = the absolute risk of preterm birth in each combination of education and mental health conditions, E- indicates high education (unexposed), and M- indicates no mental health conditions (unexposed). AP takes values between − 1 and + 1. AP > 0, AP < 0, and AP = 0 suggests positive, negative, and no additive interaction, respectively [16].

In the second interaction analysis, we examined additive interaction between age and the different combinations of education and mental health conditions. In this analysis, we calculated AP in the groups that were doubly exposed (A + EM+) to both age ≤ 23 or ≥ 31 years (A+) and each of the different exposed combinations of education and mental health conditions (EM+):

$$AP=\frac{R{R}_{A+ EM+}-R{R}_{A- EM+}-R{R}_{A+ EM-}+1}{R{R}_{A+ EM+}}=\frac{p_{A+ EM+}-{p}_{A- EM+}-{p}_{A+ EM-}+{p}_{A- EM-}}{p_{A+ EM+}}$$

A- was age 24–30 years (unexposed) and EM- was the combination of high education and no mental health conditions (unexposed).

Given that the proportion of women registered with a mental health condition increased during the study period [34], we performed supplementary analyses, including all main analyses described above for women giving birth in the periods 2000–2008 and 2009–2016, separately.

Furthermore, we performed a supplementary analysis of the risk of extreme preterm birth, defined by WHO as birth before 28 completed weeks of gestation (< 196 days) [27], because these children are facing the largest risk of death, disability and use of resources [35].

For some short-term mental health conditions, including information 5 years prior to birth might be too long. Therefore, sensitivity analyses were performed considering maternal mental health conditions 2 years instead of 5 years before the birth of the child.

Analyses were conducted using Stata version 15.1, College Station, TX, USA.

### Ethics

No ethical approval is required for register-based studies in Denmark [26].

## Results

A total of 415,523 women fulfilled the inclusion criteria (Fig. 1).

Table 1 presents the total number and percentage of women in each age stratum with different combinations of education and mental health conditions. A larger proportion of the 62,363 women aged ≤23 years had a mental health condition (minor 10.0%, moderate/severe 18.5%) compared to the 227,839 women aged 24–30 years (minor 10.8%, moderate/severe 7.8%) and the 125,321 women aged ≥31 years (minor 14.0%, moderate/severe 7.7%). In the 27,252 women aged ≤23 categorised with high/intermediate education, only 1535 (5.6%) had attained a high education corresponding to ISCED level 5.

**Table 1:** Number of women in each combination of maternal educational level and mental health conditions and percentages stratified by age group, number (%)

Maternal age, years	Educational level	Mental health condition		
		No	Minor	Moderate/severe
**≤23**^**a**^	**High/intermediate**	21,465 (34.4)	2831 (4.5)	2956 (4.7)
	**Low**	23,148 (37.1)	3382 (5.4)	8581 (13.8)
**24-30**^**a**^	**High**	92,076 (40.4)	10,964 (4.8)	5196 (2.3)
	**Intermediate**	77,807 (34.1)	10,476 (4.6)	7915 (3.5)
	**Low**	15,562 (6.8)	3078 (1.4)	4765 (2.1)
**≥31**^**a**^	**High**	61,781 (49.3)	11,027 (8.8)	5096 (4.1)
	**Intermediate**	30,621 (24.4)	5343 (4.3)	3274 (2.6)
	**Low**	5654 (4.5)	1219 (1.0)	1306 (1.0)

^a^Percentages are calculated within strata; thus, each age group sums to 100%

During the study period, 6.3% gave birth prematurely to a liveborn infant. In the women aged ≤23, 24–30, and ≥ 31 years 6.5, 6.1, and 6.7% gave birth prematurely, respectively. In all age strata, the absolute risk of preterm birth increased with decreasing educational level and increasing severity of mental health conditions (Table 2). The highest absolute risk was 12.9% [95% CI: 11.2;14.8%] in women aged ≥31 years with low education and moderate/severe mental health conditions.

**Table 2:** Absolute risk of preterm birth in each combination of maternal educational level and mental health conditions by age group, % [95% CI] (number)

Maternal age, years	Educational level	Mental health condition		
		No	Minor	Moderate/severe
**≤23**	**High/intermediate**	6.1 [5.8;6.4] (1305)	6.4 [5.5;7.3] (180)	7.0 [6.1;7.9] (206)
	**Low**	6.3 [6.0;6.6] (1460)	6.6 [5.8;7.5] (223)	7.6 [7.0;8.2] (651)
**24–30**	**High**	5.6 [5.4;5.7] (5137)	6.0 [5.6;6.5] (662)	6.5 [5.8;7.2] (336)
	**Intermediate**	6.3 [6.1;6.5] (4906)	6.8 [6.3;7.3] (714)	7.2 [6.6;7.7] (566)
	**Low**	6.3 [6.0;6.7] (986)	8.3 [7.4;9.4] (257)	8.5 [7.8;9.3] (406)
**≥31**	**High**	5.8 [5.6;6.0] (3564)	6.6 [6.2;7.1] (733)	7.6 [6.9;8.3] (385)
	**Intermediate**	7.2 [6.9;7.5] (2213)	8.1 [7.4;8.9] (434)	9.0 [8.0;10.0] (294)
	**Low**	8.4 [7.7;9.2] (477)	8.8 [7.3;10.5] (107)	12.9 [11.2;14.8] (168)

The highest relative risk in women aged ≤23, 24–30, and ≥ 31 years was found in those with low education and moderate/severe mental health conditions who had 25% [95% CI: 14–37%], 53% [95% CI: 39–68%], and 123% [95% CI: 93–158%] higher risk of preterm birth, respectively, compared to the unexposed reference groups (Table 3).

**Table 3:** Relative risk (RR) of preterm birth in each combination of maternal educational level and mental health conditions stratified by age group, RR [95% CI]

Maternal age, years	Educational level	Mental health condition		
		No	Minor	Moderate/severe
**≤23**	**High/intermediate**	1 [ref]	1.05 [0.90;1.22]	1.15 [0.99;1.32]
	**Low**	1.04 [0.97;1.12]	1.08 [0.95;1.24]	1.25 [1.14;1.37]
**24–30**	**High**	1 [ref]	1.08 [1.00;1.17]	1.16 [1.04;1.29]
	**Intermediate**	1.13 [1.09;1.17]	1.22 [1.13;1.32]	1.28 [1.18;1.39]
	**Low**	1.14 [1.06;1.21]	1.50 [1.33;1.69]	1.53 [1.39;1.68]
**≥31**	**High**	1 [ref]	1.15 [1.07;1.24]	1.31 [1.18;1.45]
	**Intermediate**	1.25 [1.19;1.32]	1.41 [1.28;1.55]	1.56 [1.39;1.74]
	**Low**	1.46 [1.33;1.60]	1.52 [1.27;1.83]	2.23 [1.93;2.58]

**Fig. 2:**
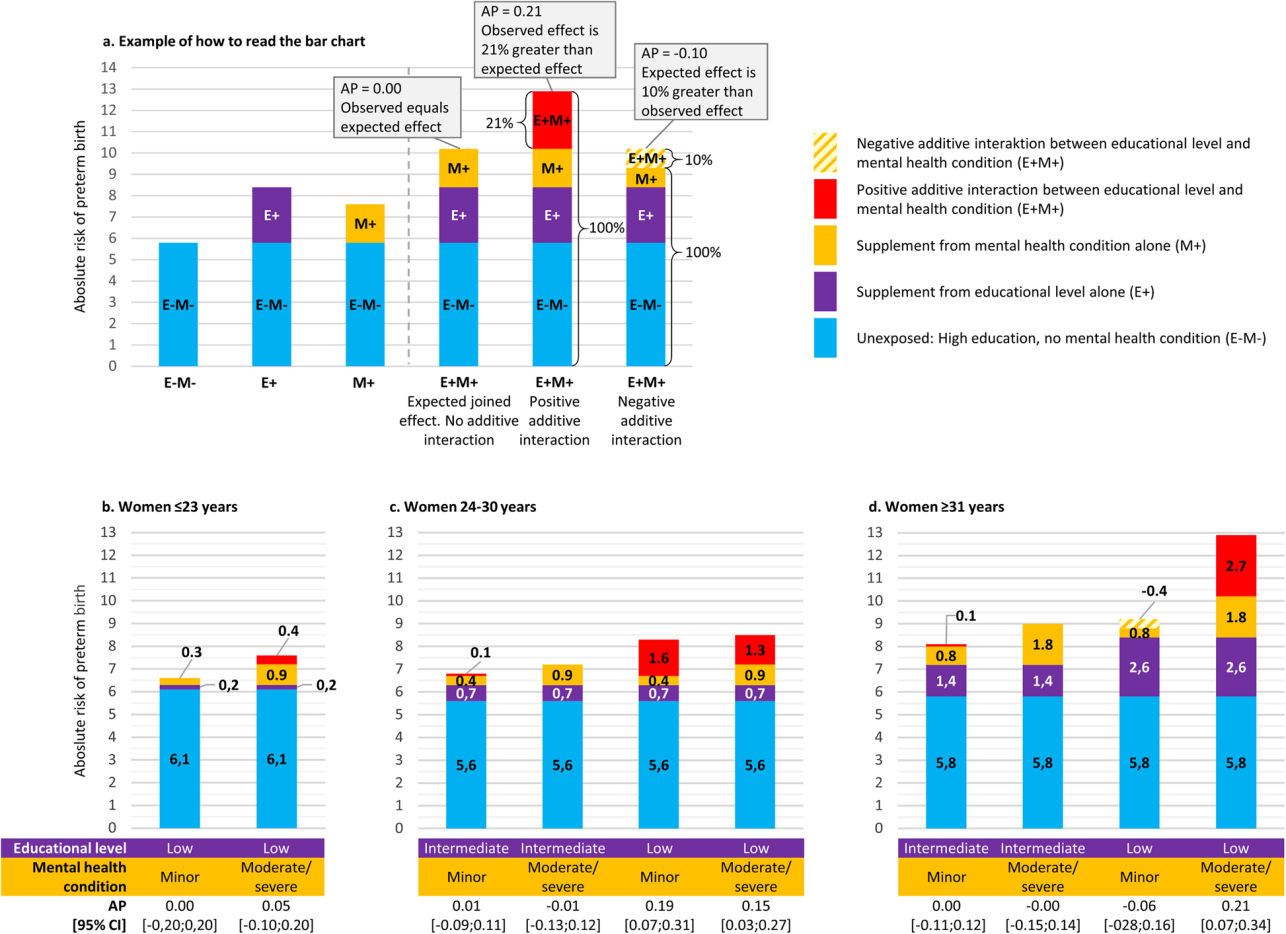
The results of the first analysis of additive interaction between educational level and mental health conditions in pregnant women aged ≤23 (**b**), 24–30 (**c**), and ≥ 31 (**d**) years. **a** illustrates an example of how to read the attributable proportion (AP) in the bar charts

In the first interaction analysis, we found positive additive interaction between low education and minor and moderate/severe mental health conditions in women aged 24–30 years (Fig. 2c) and between low education and moderate/severe mental health conditions in women aged ≥31 years (Fig. 2d). The AP in the last-mentioned group indicates that 21% [95% CI: 7;34%] of the absolute risk of preterm birth in this doubly exposed group was due to additive interaction as illustrated in Fig. 2.

**Fig. 3:**
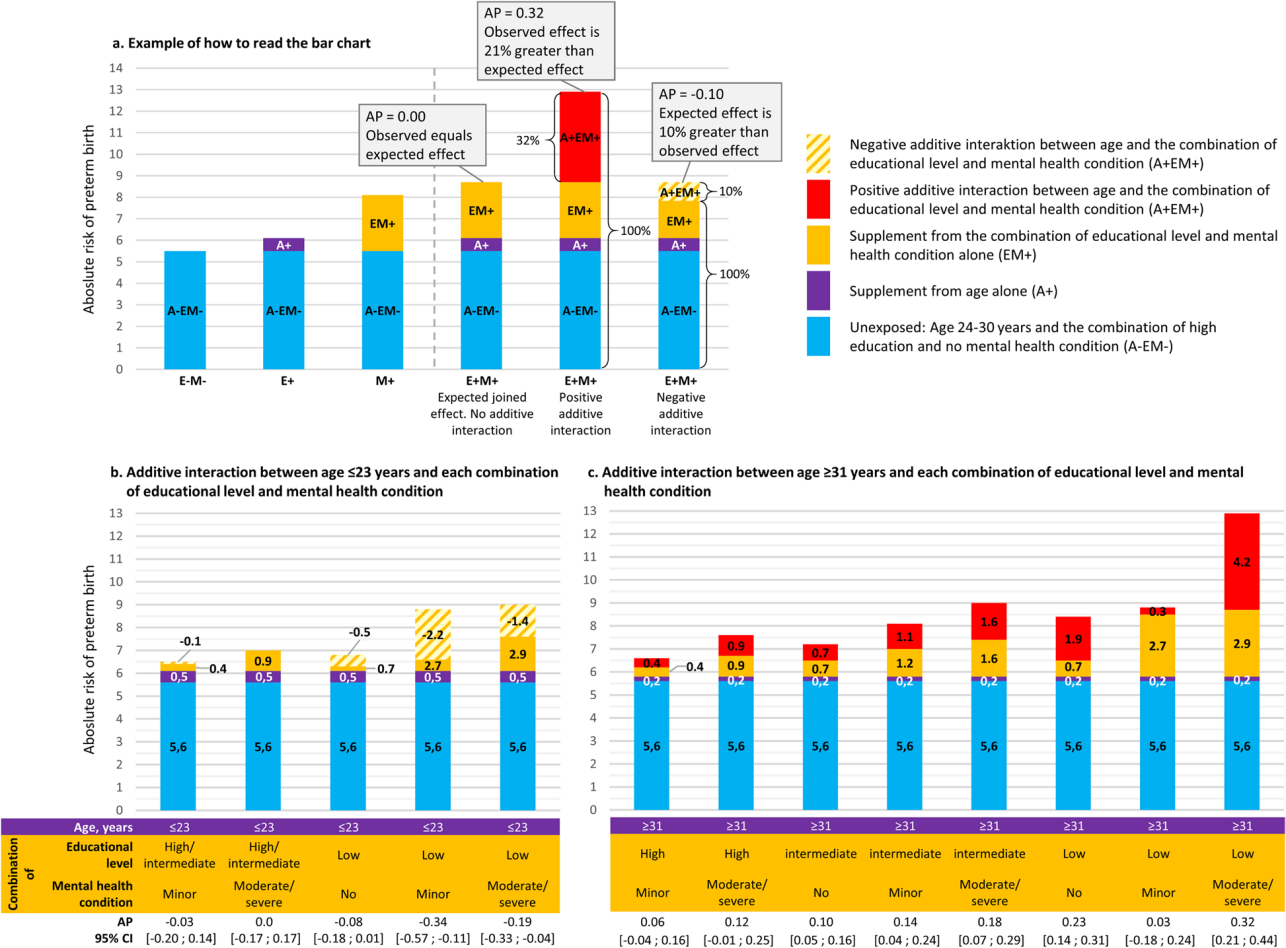
The results of the second analysis of additive interaction between age and the combinations of education and mental health conditions. **a** illustrates an example of how to read the attributable proportion (AP) in the bar charts. **b** presents the results of the analyses of additive interaction with age ≤ 23 year, and **c** presents the results of the analyses of additive interaction with age ≥ 31 years. In both (**b**) and (**c**), the unexposed group was women aged 24–30 years with the combination of high educational level and no mental health conditions

The second interaction analysis where we examined additive interaction between age and the combinations of education and mental health conditions are presented in Fig. 3. We found negative additive interaction between age ≤ 23 and the combinations of low education and no, minor, and moderate/severe mental health conditions (Fig. 3b). The expected joint effect based on summing the independent effects of both age ≤ 23 years and the combination of low education and moderate/severe mental health conditions was 19% greater than the observed effect indicated by the AP of − 0.19 [95% CI: − 0.33;-0.04]. We found positive additive interaction with age ≥ 31 years in most of the eight combinations of education and mental health conditions (Fig. 3c). For women with the combined exposure of low education and moderate/severe mental health conditions, further being exposed to age ≥ 31 resulted in an AP of 0.32 [95% CI: 0.21;0.44%] indicating that 32% of the risk of preterm birth among these women could be explained by the interaction itself.

The supplementary analyses stratified by study period are presented in Supplementary tables s4-s14 [Additional file 1]. Overall, the risk of preterm birth decreased from 6.5% [95% CI: 6.4;6.6%] in 2000–2008 to 6.1% [95% CI: 6.0;6.2%] in 2009–2016, corresponding to a decrease in the overall risk in all three age strata (see Supplementary tables s8 in Additional file 1). However, the relative risks and the AP measures did not vary remarkably between the first and the last part of the study period.

The supplementary analysis of the risk of extreme preterm birth showed to some extent similar patterns of the risk increasing with lower educational level and the severity of mental health conditions, see Supplementary tables s15-s16 [Additional file 1]. In general, for women aged ≥24 years the relative risks were higher than in the main analyses. However, there were a limited number of extreme preterm births in our study population, resulting in very broad confidence intervals.

Our sensitivity analyses, where we considered maternal mental health conditions 2 years instead of 5 years before the birth of the child, showed increased risks for the women with mental health conditions (see Supplementary tables s17-s21 [Additional file 1]). However, the AP measures were similar to the AP measures in the main analyses.

## Discussion

In this study, we found that the risk of preterm birth increased with decreasing educational level and increasing severity of mental health conditions in all age groups. However, this inequality increased substantially with increasing age. The positive additive interaction between low education and mental health conditions in women aged 24–30 and ≥ 31 years, found in the first interaction analysis, indicates a higher impact on the risk of preterm birth when doubly exposed in these two age groups.

The second interaction analysis of the additive interaction between age and the combinations of education and mental health conditions revealed negative additive interaction with age ≤ 23 years and positive additive interaction with age ≥ 31 years. This indicates that with increasing age, the impact of education and mental health conditions, both separately and in combination, are more consequential to the risk of preterm birth. The results from this interaction analysis further suggests that when resources are limited, intervention strategies may have the potential to prevent a larger proportion of preterm births if targeting women with higher age further combined with lower educational levels and mental health conditions.

In 2009, a shift was seen in the national Danish antenatal guidelines from universal towards differentiated services, as recommended by WHO [36], with the intention of giving greater priority to disadvantaged pregnant women [37]. Potentially, this differentiated antenatal care could have reduced the inequality in preterm birth. However, our supplementary analyses revealed that inequality remained unaffected before and after 2009, despite the decrease in the overall risk of preterm birth.

The direction of increasing risk of preterm birth in women with mental health conditions and/or decreasing educational level found in this study is consistent with other Danish and international studies examining the exposures separately [3, 6, 7, 9, 38]. However, the suggested mechanisms underlying the inequality in preterm birth are complex and not fully understood [39, 40]. Often, inequality in preterm birth has been attributed to socially patterned lifestyle [41]. However, lifestyle factors such as smoking, alcohol consumption, and body mass index, only explain a small part of the educational disparities in preterm birth [6, 41]. Studies have found that preterm birth is associated with psychological and social stress [42, 43] which is considered leading to preterm birth through neuroendocrine, inflammatory, and immunological mechanisms [39, 42]. Socially disadvantaged pregnant women may lead more stressful lives due to exposure to stressors such as unemployment, financial hardship, discrimination, unstable social relations, and lack of social support [40, 44]. The combination of low or intermediate education and mental health conditions could entail further accumulation of stressors compared to women separately exposed and therefore explain some of the higher risk of preterm birth.

It is suggested that the risk of negative birth outcomes increases with longer duration of exposure to risk factors and that repeated exposure to stressors could increase the risk of preterm birth [45]. Longer duration could explain some of the more consequential impact on preterm birth of low education and mental health conditions for pregnant women at advancing age found in this study.

### Strengths and limitations

There are several strengths to this study. The national Danish registers contain high-quality data covering the entire population [26]. This resulted in a nationwide cohort which enabled examination of the risk of preterm birth in numerous combinations of educational level, mental health conditions, and age allowing for identification of relatively specific high-risk subgroups. Minor mental health conditions are often undiagnosed [46] and therefore not included in register-based studies. By including medication and contact to general practitioners and private psychologists, we were able to identify pregnant women with minor mental health conditions which we found had a noteworthy higher risk of preterm birth.

There are also some limitations to this study. We only identified mental health conditions of women who sought medical care and were registered with contact to the primary or secondary healthcare system or redeemed prescriptions within a window of 5 years before the birth of the child. Therefore, some women with mental health conditions might have been misclassified with no mental health conditions. The consideration of mental health conditions in a window of 5 years may also have classified some women as having a mental health condition at childbirth even though they were actually recovered. Accordingly, our sensitivity analyses of mental health conditions considered 2 years instead of 5 years before childbirth showed slightly increased risks for the women with mental health conditions. However, this finding did not change the overall interpretation of the study results.

Although misclassification of the highest educational level attained is unlikely due to mandatory registration of completed education by the educational institutions [25], the merging of high and intermediate education in women aged ≤23 years might have led to bias towards the null. However, the proportion of women who had reached a high education within this age group was limited.

Stillbirths is associated with preterm birth [47] but were not included in this study. Though stillbirths do not count numerous births in Denmark [48], we may have underestimated the true burden of the inequality in preterm birth by not including stillbirths, which are inversely associated with maternal educational level [7].

Due to the exclusion criteria, the results might not be generalisable to women with multiple pregnancies, as these women have an increased risk of preterm birth, and to multiparous pregnant women, because previous preterm birth increases the risk in later pregnancy [5]. The women excluded due to the criteria of a Danish registered address, are likely to be recent immigrants. Hence, the results might not be generalisable to all immigrant pregnant women.

### Implication of findings

The substantial increased risk of preterm birth found in women with combinations of decreasing educational level and increasing severity of mental health conditions emphasises the importance of identification of these disadvantaged pregnant women in the antenatal care to reduce the inequality in preterm birth. Routine antenatal psychosocial risk assessment may increase awareness of these psychosocial risks [49]. Our results indicate that such psychosocial assessment should not neglect minor mental health conditions. Systematic screening during pregnancy is important to ensure that pregnant women with mental health conditions and lower educational levels are referred to relevant, tailored services and that such specialized, supportive interventions are freely available to women, based on their individual needs.

In many countries, public health policy has focused attention on younger mothers and their adverse perinatal outcomes [10]. However, despite the larger proportion of mental health conditions in women aged ≤23 years our findings reveal that intervention strategies with the purpose of reducing inequality in preterm birth should target women with higher age further combined with lower educational levels and mental health conditions, especially when resources are limited.

That the inequality remained unaffected from the first to the last part of the study period despite greater priority to disadvantaged pregnant women implicates that improved intervention strategies are needed targeting disadvantaged pregnant women with lower educational levels and mental health conditions, and particularly those aged ≥31 years.

Further studies are needed to examine variables that drive the heterogeneity across the social positions found in this study in order to improve intervention strategies targeting disadvantaged pregnant women with lower educational levels and mental health conditions.

## Conclusion

Substantial inequality in preterm birth remains with increasing risk in disadvantaged pregnant women with decreasing educational level and increasing severity of mental health conditions. The inequality in preterm birth increased with increasing age. Thus, more awareness of women with higher age further combined with lower educational levels and mental health conditions is needed in the prevention of the inequality in preterm birth.

## Supplementary Information

**Additional file 1: Supplementary Table s1.** Definition of the outcome preterm birth. **Supplementary Table s2.** Definition of educational levels. **Supplementary Table s3.** Definition of mental health conditions. **Supplementary Table s4.** Number in each combination of maternal educational level and mental health condition in the period 2000–2008 and percentages stratified by age group, number (%). **Supplementary Table s5.** Number in each combination of maternal educational level and mental health condition in the period 2009–2016 and percentages stratified by age group, number (%). **Supplementary Table s6.** Absolute risk of preterm birth in the period 2000–2008 in each combination of maternal educational level and mental health condition by age group, % [95% CI] (number). **Supplementary Table s7.** Absolute risk of preterm birth in the period 2009–2016 in each combination of maternal educational level and mental health condition by age group, % [95% CI] (number). **Supplementary Table s8.** Overall risk of preterm birth in the three age strata in the periods 2000–2008 and 2009–2016, % [CI 95%]. **Supplementary Table s9.** Relative risk [RR] of preterm birth in the period 2000–2008 in each combination of maternal educational level and mental health condition stratified by age group, RR [95% CI]. **Supplementary Table s10.** Relative risk [RR] of preterm birth in the period 2009–2016 in each combination of maternal educational level and mental health condition stratified by age group, RR [95% CI]. **Supplementary Table s11.** Additive interaction between educational level and mental health conditions stratified by age group measured as the attributable proportion (AP) of the risk in 2000–2008 in the groups that are doubly exposed to both intermediate or low educational level and minor or moderate/severe mental health condition, AP [95% CI]. **Supplementary Table s12.** Additive interaction between educational level and mental health conditions stratified by age group measured as the attributable proportion (AP) of the risk in 2009–2016 in the groups that are doubly exposed to both intermediate or low educational level and minor or moderate/severe mental health condition, AP [95% CI]. **Supplementary Table s13.** Attributable proportion (AP) of the risk in 2000–2008 in the group that is doubly exposed to both age ≤ 23 or ≥ 31 years and each combination of educational level and mental health conditions, AP [95% CI]. **Supplementary Table s14.** Attributable proportion (AP) of the risk in 2009–2016 in the group that is doubly exposed to both age ≤ 23 or ≥ 31 years and each combination of educational level and mental health conditions, AP [95% CI]. **Supplementary Table s15.** Absolute risk of extreme preterm birth (< 28 weeks) in each combination of maternal educational level and mental health condition by age group, % [95% CI] (number). **Supplementary Table s16.** Relative risk [RR] of extreme preterm birth (< 28 weeks) in each combination of maternal educational level and mental health condition stratified by age group, RR [95% CI]. **Supplementary Table s17.** Number of women in each combination of maternal educational level and mental health condition considered 2 years before childbirth and percentages stratified by age group, number (%). **Supplementary Table s18.** Absolute risk of preterm birth in each combination of maternal educational level and mental health condition considered 2 years before childbirth by age group, % [95% CI] (number). **Supplementary Table s19.** Relative risk [RR] of preterm birth in each combination of maternal educational level and mental health condition considered 2 years before childbirth stratified by age group, RR [95% CI]. **Supplementary Table s20.** Additive interaction between educational level and mental health conditions considered 2 years before childbirth stratified by age group measured as the attributable proportion (AP) of the risk in the groups that are doubly exposed to both intermediate or low educational level and minor or moderate/severe mental health condition, AP [95% CI]. **Supplementary Table s21:** Attributable proportion (AP) of the risk in the group that is doubly exposed to both age ≤ 23 or ≥ 31 years and each combination of educational level and mental health conditions considered 2 years before childbirth, AP [95% CI].

## Data Availability

The data that support the findings of this study is available from Statistics Denmark, but restrictions apply to the availability of these data, which were used under license for the current study and so are not publicly available. Questions or requests concerning this data is directed to the corresponding author Camilla Klinge Knudsen.
