# Supplementary material for: The risk of preterm birth in combinations of socioeconomic position and mental health conditions in different age groups: a Danish nationwide register-based cohort study
Source: BMC Pregnancy Childbirth. 2021 Oct 14;21:696. doi: 10.1186/s12884-021-04138-0 (PMC8515728; doi:10.1186/s12884-021-04138-0)
Supplement: Supplementary file 1 — Additional file 1: Supplementary Table s1. Definition of the outcome preterm birth. Supplementary Table s2. Definition of educational levels. Supplementary Table s3. Definition of mental health conditions. Supplementary Table s4. Number in each combination of maternal educational level and mental health condition in the period 2000–2008 and percentages stratified by age group, number (%). Supplementary Table s5. Number in each combination of maternal educational level and mental health condition in the period 2009–2016 and percentages stratified by age group, number (%). Supplementary Table s6. Absolute risk of preterm birth in the period 2000–2008 in each combination of maternal educational level and mental health condition by age group, % [95% CI] (number). Supplementary Table s7. Absolute risk of preterm birth in the period 2009–2016 in each combination of maternal educational level and mental health condition by age group, % [95% CI] (number). Supplementary Table s8. Overall risk of preterm birth in the three age strata in the periods 2000–2008 and 2009–2016, % [CI 95%]. Supplementary Table s9. Relative risk [RR] of preterm birth in the period 2000–2008 in each combination of maternal educational level and mental health condition stratified by age group, RR [95% CI]. Supplementary Table s10. Relative risk [RR] of preterm birth in the period 2009–2016 in each combination of maternal educational level and mental health condition stratified by age group, RR [95% CI]. Supplementary Table s11. Additive interaction between educational level and mental health conditions stratified by age group measured as the attributable proportion (AP) of the risk in 2000–2008 in the groups that are doubly exposed to both intermediate or low educational level and minor or moderate/severe mental health condition, AP [95% CI]. Supplementary Table s12. Additive interaction between educational level and mental health conditions stratified by age group measured as the attributable proport [file 12884_2021_4138_MOESM1_ESM.pdf]

BMC Pregnancy and Childbirth

The risk of preterm birth in combinations of socioeconomic position and mental health conditions in different age groups: A Danish nationwide register-based cohort study

- Additional file 1 - Supplementary tables

Camilla Klinge Knudsen*[1,2]

Amanda Marie Somer Christesen[1,2]

Signe Heuckendorff[1,3]

Kirsten Fonager[1,4]

Martin Nygård Johansen[5]

Charlotte Overgaard[2]

[1] Department of Social Medicine, Aalborg University Hospital, Aalborg, Denmark

[2] Public Health and Epidemiology Group, Department of Health Science and Technology, Aalborg University, Aalborg, Denmark

[3] Danish Center for Clinical Health Services Research (DACS), Department of Clinical Medicine, Aalborg University, Aalborg, Denmark

[4] Department of Clinical Medicine, Aalborg University, Aalborg, Denmark

[5] Unit of Clinical Biostatistics, Aalborg University Hospital, Aalborg, Denmark

*Corresponding author: Camilla Klinge Knudsen, ca-klinge@live.dk

Supplementary table of contents:

Supplementary tables s1-s3: Definition of variables

Supplementary tables s4-s14: Supplementary analyses of all main analyses in the periods 2000-2008 and 2009-2016 separately

Supplementary tables s15-s16: Supplementary analysis of the risk of extreme preterm birth (<28 weeks)

Supplementary tables s17-s21: Sensitivity analyses considering maternal mental health conditions two years prior to birth of the child.

Supplementary tables s1-s3: Definition of variables

Supplementary table s1: Definition of the outcome preterm birth

Outcome	Registry	Description
**Preterm birth**	The Danish Medical Birth Register (MFR)	Preterm birth was defined as birth before 37 completed weeks of gestation, that is birth before 259 days of gestational age

Supplementary table s2: Definition of educational levels

Educational level	Registry	Description
**Low education**	Statistics Denmark's registry on education (UDDA)	**ISCED Levels 0-2:** Early childhood education, primary education and lower secondary education corresponding to 1-10 years of education
**Intermediate education**	Statistics Denmark's registry on education (UDDA)	**ISCED Levels 3-4:** General upper secondary education, high school programs, vocational upper secondary education, vocational training, and education corresponding to approximately 10-12 years of education
**High education**	Statistics Denmark's registry on education (UDDA)	**ISCED Levels 5-8:** Short, medium-length or long length higher education, first, second or third cycle programs, tertiary education, bachelor or equivalent, Master's or equivalent, Doctoral, PhD programs or equivalent corresponding to more than approximately 12 years of education

Supplementary table s3: Definition of mental health conditions

Mental health conditions were grouped in three mutually exclusive severity groups in a spectrum from severe mental health conditions to minor mental health conditions. All variables are measured within 5 years before and until the birth of the child.

**Mental health conditions**	**Registry**	**Diagnosis or codes** (and/or)	**Description**
**Moderate/severe mental health conditions**	Danish National Patient Registry	Any psychiatric diagnosis (ICD-10: F00-99) registered at a psychiatric hospital (In- and outpatient contacts, primary and secondary diagnosis)	Handled at psychiatric hospital or private psychiatrist
	Danish National Health Service Register: Private psychiatrist	One or more *contacts* with a private psychiatrist	
**Minor mental health conditions**	National Health Service Prescriptions Database	*At least two* redeemed prescriptions of antidepressants or benzodiazepines <5 years before birth of the child<br><br>ATC-codes:<br>Benzodiazepines: MN03AE, MN05BA, MN05CD, MN05CF<br>SSRI: N06AB<br>Other antidepressants: N06AX	Only handled in the primary healthcare sector (general practice and/or private psychologist) without any psychiatric hospital contacts or contacts to private psychiatrists.
	Danish National Health Service Register: Psychologist	One or more *contacts* with a psychologist	
	Danish National Health Service Register: General practitioner	Talk therapy, at least two sessions<br><br>Psychometric tests, at least two	
**No mental health conditions**		None of the above	

Supplementary tables s4-14: Supplementary analyses of all main analyses in the periods 2000-2008 and 2009-2016 separately

Supplementary table s4:

Supplementary table s4: Number in each combination of maternal educational level and mental health condition in the period 2000-2008 and percentages stratified by age group, number (%)

Maternal age, years	Educational level	Mental health condition		
		No	Minor	Moderate/severe
≤23*	High/intermediate	11,829 (35.1)	911 (2.7)	1,174 (3.5)
	Low	14,662 (43.5)	1,409 (4.2)	3,685 (10.9)
24-30*	High	48,772 (38.5)	3,697 (2.9)	2,147 (1.7)
	Intermediate	50,838 (40.1)	4,086 (3.2)	3,698 (2.9)
	Low	10,125 (8.0)	1,275 (1.0)	2,008 (1.6)
≥31*	High	30,552 (47.9)	3,740 (5.9)	2,006 (3.1)
	Intermediate	18,917 (29.7)	2,263 (3.6)	1,499 (2.4)
	Low	3,632 (5.7)	545 (0.9)	578 (0.9)

**Percentages are calculated within strata; thus, each age group sums to 100%*

Supplementary table s5:

Supplementary table s5: Number in each combination of maternal educational level and mental health condition in the period 2009-2016 and percentages stratified by age group, number (%)

Maternal age, years	Educational level	Mental health condition		
		No	Minor	Moderate/severe
≤23*	High/intermediate	9,636 (33.6)	1,920 (6.7)	1,782 (6.2)
	Low	8,486 (29.6)	1,973 (6.9)	4,896 (17.1)
24-30*	High	43,304 (42.8)	7,267 (7.2)	3,049 (3.0)
	Intermediate	26,969 (26.7)	6,390 (6.3)	4,217 (4.2)
	Low	5,437 (5.4)	1,803 (1.8)	2,757 (2.7)
≥31*	High	31,229 (50.7)	7,287 (11.8)	3,090 (5.0)
	Intermediate	11,704 (19.0)	3,080 (5.0)	1,775 (2.9)
	Low	2,022 (3.3)	674 (1.1)	728 (1.2)

**Percentages are calculated within strata; thus, each age group sums to 100%*

Supplementary table s6:

Supplementary table s6: Absolute risk of preterm birth in the period 2000-2008 in each combination of maternal educational level and mental health condition by age group, % [95% CI] (number)

Maternal age, years	Educational level	Mental health condition		
		No	Minor	Moderate/severe
≤23	High/intermediate	6.6 [6.1;7.0] (777)	8.3 [6.7;10.3] (76)	7.6 [6.2;9.2] (89)
	Low	6.5 [6.1;6.9] (954)	7.5 [6.3;9.0] (106)	7.3 [6.5;8.2] (270)
24-30	High	5.7 [5.5;5.9] (2,799)	6.7 [5.9;7.5] (246)	6.5 [5.6;7.6] (140)
	Intermediate	6.3 [6.1;6.5] (3,219)	6.9 [6.1;7.7] (280)	7.5 [6.7;8.3] (278)
	Low	6.7 [6.2;7.2] (677)	9.7 [8.2;11.5] (124)	8.8 [7.7;10.1] (177)
≥31	High	6.0 [5.8;6.3] (1,839)	7.4 [6.6;8.3] (277)	7.8 [6.7;9.0] (156)
	Intermediate	7.4 [7.1;7.8] (1,405)	8.8 [7.7;10.0] (199)	8.3 [7.0;9.8] (124)
	Low	8.5 [7.7;9.5] (310)	9.4 [7.2;12.1] (51)	13.1 [10.6;16.2] (76)

Supplementary table s7:

Supplementary table s7: Absolute risk of preterm birth in the period 2009-2016 in each combination of maternal educational level and mental health condition by age group, % [95% CI] (number)

Maternal age, years	Educational level	Mental health condition		
		No	Minor	Moderate/severe
≤23	High/intermediate	5.5 [5.0;6.0] (528)	5.4 [4.5;6.5] (104)	6.6 [5.5;7.8] (117)
	Low	6.0 [5.5;6.5] (506)	5.9 [5.0;7.1] (117)	7.8 [7.1;8.6] (381)
24-30	High	5.4 [5.2;5.6] (2,338)	5.7 [5.2;6.3] (416)	6.4 [5.6;7.4] (196)
	Intermediate	6.3 [6.0;6.6] (1,687)	6.8 [6.2;7.4] (434)	6.8 [6.1;7.6] (288)
	Low	5.7 [5.1;6.3] (309)	7.4 [6.3;8.7] (133)	8.3 [7.3;9.4] (229)
≥31	High	5.5 [5.3;5.8] (1,725)	6.3 [5.7;6.8] (456)	7.4 [6.5;8.4] (229)
	Intermediate	6.9 [6.5;7.4] (808)	7.6 [6.7;8.6] (235)	9.6 [8.3;11.0] (170)
	Low	8.3 [7.1;9.5] (167)	8.3 [6.4;10.6] (56)	12.6 [10.4;15.3] (92)

Supplementary table s8:

Supplementary table s8: Overall risk of preterm birth in the three age strata in the periods 2000-2008 and 2009-2016, % [CI 95%]

Age group, years	Absolute risk (2000-2008)	Absolute risk (2009-2016)
**≤23**	6.7 [6.5;7.0]	6.1 [5.8;6.4]
**24-30**	6.3 [6.1;6.4]	6.0 [5.8;6.1]
**≥31**	7.0 [6.8;7.2]	6.4 [6.2;6.6]

Supplementary table s9:

Supplementary table s9: Relative risk [RR] of preterm birth in the period 2000-2008 in each combination of maternal educational level and mental health condition stratified by age group, RR [95% CI]

Maternal age, years	Educational level	Mental health condition		
		No	Minor	Moderate/severe
≤23	High/intermediate	1 [ref]	1.27 [1.01;1.59]	1.15 [0.93;1.43]
	Low	0.99 [0.90;1.09]	1.15 [0.94;1.39]	1.12 [0.98;1.27]
24-30	High	1 [ref]	1.16 [1.02;1.32]	1.14 [0.96;1.34]
	Intermediate	1.10 [1.05;1.16]	1.19 [1.06;1.34]	1.31 [1.16;1.47]
	Low	1.17 [1.07;1.26]	1.69 [1.43;2.01]	1.54 [1.33;1.78]
≥31	High	1 [ref]	1.23 [1.09;1.39]	1.29 [1.10;1.51]
	Intermediate	1.23 [1.15;1.32]	1.46 [1.27;1.68]	1.37 [1.15;1.64]
	Low	1.42 [1.26;1.59]	1.55 [1.19;2.03]	2.18 [1.76;2.71]

Supplementary table s10:

Supplementary table s10: Relative risk [RR] of preterm birth in the period 2009-2016 in each combination of maternal educational level and mental health condition stratified by age group, RR [95% CI]

Maternal age, years	Educational level	Mental health condition		
		No	Minor	Moderate/severe
≤23	High/intermediate	1 [ref]	0.99 [0.81;1.21]	1.20 [0.99;1.45]
	Low	1.09 [0.97;1.22]	1.08 [0.89;1.31]	1.42 [1.25;1.61]
24-30	High	1 [ref]	1.06 [0.96;1.17]	1.19 [1.03;1.37]
	Intermediate	1.16 [1.09;1.23]	1.26 [1.14;1.39]	1.26 [1.12;1.42]
	Low	1.05 [0.94;1.18]	1.37 [1.15;1.62]	1.54 [1.35;1.75]
≥31	High	1 [ref]	1.13 [1.03;1.25]	1.34 [1.17;1.53]
	Intermediate	1.25 [1.15;1.36]	1.38 [1.21;1.57]	1.73 [1.49;2.01]
	Low	1.50 [1.28;1.74]	1.50 [1.17;1.94]	2.29 [1.88;2.78]

Supplementary table s11:

Supplementary table s11: Additive interaction between educational level and mental health conditions stratified by age group measured as the attributable proportion (AP) of the risk in 2000-2008 in the groups that are doubly exposed to both intermediate or low educational level and minor or moderate/severe mental health condition, AP [95% CI]

Maternal age, years	Educational level	Mental health condition		
		No	Minor	Moderate/severe
≤23	High/intermediate			
	Low		-0.10 [-0.42;0.22]	-0.03 [-0.28;0.23]
24-30	High			
	Intermediate		-0.06 [-0.23;0.12]	0.05 [-0.13;0.23]
	Low		0.22 [0.05;0.38]	0.15 [-0.03;0.33]
≥31	High			
	Intermediate		-0.00 [-0.17;0.17]	-0.11 [-0.35;0.13]
	Low		-0.06 [-0.37;0.25]	0.22 [0.02;0.42]

Supplementary table s12:

Supplementary table s12: Additive interaction between educational level and mental health conditions stratified by age group measured as the attributable proportion (AP) of the risk in 2009-2016 in the groups that are doubly exposed to both intermediate or low educational level and minor or moderate/severe mental health condition, AP [95% CI]

Maternal age, years	Educational level	Mental health condition		
		No	Minor	Moderate/severe
≤23	High/intermediate			
	Low		0.01 [-0.26;0.27]	0.09 [-0.10;0.29]
24-30	High			
	Intermediate		0.03 [-0.10;0.16]	-0.07 [-0.25;0.12]
	Low		0.19 [0.01;0.36]	0.19 [0.03;0.36]
≥31	High			
	Intermediate		-0.00 [-0.16;0.16]	0.08 [-0.09;0.25]
	Low		-0.08 [-0.40;0.23]	0.20 [0.00;0.39]

Supplementary table s13:

Supplementary table s13: Attributable proportion (AP) of the risk in 2000-2008 in the group that is doubly exposed to both age≤23 or ≥31 years and each combination of educational level and mental health conditions, AP [95% CI]

Educational level	Mental health condition	Maternal age, years		
		24-30	≤23	≥31
High*	No			
	Minor		0.10 [-0.12;0.33]	0.06 [-0.09;0.22]
	Moderate or severe		0.03 [-0.22;0.28]	0.13 [-0.07;0.32]
Intermediate	No			0.11 [0.04;0.18]
	Minor			0.19 [0.04;0.33]
	Moderate or severe			0.06 [-0.14;0.25]
Low	No		-0.16 [-0.28;-0.03]	0.18 [0.07;0.29]
	Minor		-0.40 [-0.75;-0.06]	-0.07 [-0.40;0.26]
	Moderate or severe		-0.32 [-0.55;-0.08]	0.31 [0.13;0.48]

**For women aged≤23 years high and intermediate educational level was merged*

Supplementary table s14:

Supplementary table s14: Attributable proportion (AP) of the risk in 2009-2016 in the group that is doubly exposed to both age≤23 or ≥31 years and each combination of educational level and mental health conditions, AP [95% CI]

Educational level	Mental health condition	Maternal age, years		
		24-30	≤23	≥31
High*	No			
	Minor		-0.07 [-0.31;0.17]	0.07 [-0.07;0.20]
	Moderate or severe		0.01 [-0.22;0.24]	0.12 [-0.05;0.28]
Intermediate	No			0.08 [-0.01;0.16]
	Minor			0.09 [-0.05;0.24]
	Moderate or severe			0.27 [0.14;0.41]
Low	No		0.03 [-0.12;0.19]	0.30 [0.16;0.43]
	Minor		-0.26 [-0.57;0.05]	0.10 [-0.17;0.37]
	Moderate or severe		-0.08 [-0.26;0.10]	0.33 [0.18;0.49]

**For women aged≤23 years high and intermediate educational level was merged*

Supplementary tables s15-s16: Supplementary analysis of the risk of extreme preterm birth (<28 weeks)

Supplementary table s15

Supplementary table s15: Absolute risk of extreme preterm birth (<28 weeks) in each combination of maternal educational level and mental health conditions by age group, % [95% CI] (number)

Maternal age, years	Educational level	Mental health condition		
		No	Minor	Moderate/severe
≤23	High/intermediate	0.4 [0.3;0.4] (77)	0.3 [0.2;0.6] (9)	0.2 [0.1;0.5] (6)
	Low	0.3 [0.3;0.4] (79)	0.4 [0.2;0.6] (12)	0.5 [0.4;0.7] (43)
24-30	High	0.2 [0.2;0.2] (176)	0.2 [0.1;0.3] (24)	0.2 [0.1;0.4] (11)
	Intermediate	0.3 [0.2;0.3] (202)	0.4 [0.3;0.5] (37)	0.4 [0.3;0.6] (31)
	Low	0.4 [0.3;0.5] (61)	0.5 [0.3;0.8] (16)	0.5 [0.3;0.7] (23)
≥31	High	0.3 [0.3;0.4] (213)	0.4 [0.3;0.5] (44)	0.4 [0.3;0.6] (20)
	Intermediate	0.4 [0.4;0.5] (136)	0.8 [0.6;1.0] (41)	0.6 [0.4;0.9] (19)
	Low	0.7 [0.5;0.9] (39)	0.7 [0.4;1.4] (9)	0.8 [0.4;1.4] (10)

Supplementary table s16

Supplementary table s16: Relative risk (RR) of extreme preterm birth (<28 weeks) in each combination of maternal educational level and mental health conditions stratified by age group, RR [95% CI]

Maternal age, years	Educational level	Mental health condition		
		No	Minor	Moderate/severe
≤23	High/intermediate	1 [ref]	0.89 [0.44;1.77]	0.57 [0.25;1.30]
	Low	0.95 [0.70;1.30]	0.99 [0.54;1.82]	1.40 [0.96;2.03]
24-30	High	1 [ref]	1.15 [0.75;1.75]	1.11 [0.60;2.04]
	Intermediate	1.36 [1.11;1.66]	1.85 [1.30;2.63]	2.05 [1.40;3.00]
	Low	2.05 [1.53;2.74]	2.72 [1.63;4.53]	2.53 [1.64;3.90]
≥31	High	1 [ref]	1.16 [0.84;1.60]	1.14 [0.72;1.80]
	Intermediate	1.29 [1.04;1.60]	2.23 [1.60;3.11]	1.68 [1.05;2.69]
	Low	2.00 [1.42;2.81]	2.14 [1.10;4.16]	2.22 [1.18;4.18]

Supplementary tables s17-s21: Sensitivity analyses considering maternal mental health conditions two years prior to birth of the child.

Supplementary table s17

Supplementary table s17: Number of women in each combination of maternal educational level and mental health conditions concidered two years before childbirth and percentages stratified by age group, number (%)

Maternal age, years	Educational level	Mental health condition (concidered two years before childbirth)		
		No	Minor	Moderate/severe
≤23*	High/intermediate	23,862 (38.3)	1,789 (2.9)	1,601 (2.6)
	Low	26,915 (43.2)	2,541 (4.1)	5,655 (9.1)
24-30*	High	99,281 (43.6)	6,116 (2.7)	2,839 (1.2)
	Intermediate	85,566 (37.6)	6,148 (2.7)	4,484 (2.0)
	Low	18,401 (8.1)	2,045 (0.9)	2,959 (1.3)
≥31*	High	68,485 (54.6)	6,211 (5.0)	3,208 (2.6)
	Intermediate	34,193 (27.3)	3,048 (2.4)	1,997 (1.6)
	Low	6,521 (5.2)	797 (0.6)	861 (0.7)

**Percentages are calculated within strata; thus, each age group sums to 100%*

Supplementary table s18

Supplementary table s18: Absolute risk of preterm birth in each combination of maternal educational level and mental health conditions concidered two years before childbirth by age group, % [95% CI] (number)

Maternal age, years	Educational level	Mental health condition (concidered two years before childbirth)		
		No	Minor	Moderate/severe
≤23	High/intermediate	6.0 [5.7;6.3] (1,434)	7.4 [6.3;8.7] (132)	7.8 [6.6;9.2] (125)
	Low	6.3 [6.0;6.6] (1,688)	6.9 [6.0;8.0] (176)	8.3 [7.6;9.1] (470)
24-30	High	5.6 [5.4;5.7] (5,527)	6.6 [6.0;7.2] (403)	7.2 [6.3;8.2] (205)
	Intermediate	6.3 [6.1;6.5] (5,389)	7.1 [6.5;7.8] (438)	8.0 [7.2;8.8] (359)
	Low	6.5 [6.1;6.8] (1,191)	8.7 [7.5;10.0] (177)	9.5 [8.5;10.6] (281)
≥31	High	5.8 [5.6;6.0] (3,972)	7.1 [6.5;7.8] (442)	8.4 [7.4;9.4] (268)
	Intermediate	7.3 [7.0;7.6] (2,486)	8.5 [7.6;9.5] (259)	9.8 [8.6;11.2] (196)
	Low	8.5 [7.9;9.2] (555)	10.9 [8.9;13.3] (87)	12.8 [10.7;15.2] (110)

Supplementary table s19

Supplementary table s19: Relative risk (RR) of preterm birth in each combination of maternal educational level and mental health conditions concidered two years before childbirth stratified by age group, RR [95% CI]

Maternal age, years	Educational level	Mental health condition (concidered two years before childbirth)		
		No	Minor	Moderate/severe
≤23	High/intermediate	1 [ref]	1.23 [1.03;1.46]	1.30 [1.09;1.55]
	Low	1.04 [0.97;1.12]	1.15 [0.99;1.34]	1.38 [1.25;1.53]
24-30	High	1 [ref]	1.18 [1.07;1.31]	1.30 [1.13;1.48]
	Intermediate	1.13 [1.09;1.17]	1.28 [1.17;1.41]	1.44 [1.30;1.59]
	Low	1.16 [1.09;1.24]	1.55 [1.35;1.79]	1.71 [1.52;1.91]
≥31	High	1 [ref]	1.23 [1.12;1.35]	1.44 [1.28;1.62]
	Intermediate	1.25 [1.19;1.32]	1.47 [1.30;1.65]	1.69 [1.48;1.94]
	Low	1.47 [1.35;1.60]	1.88 [1.54;2.30]	2.20 [1.85;2.63]

Supplementary table s20

Supplementary table s20: Additive interaction between educational level and mental health conditions concidered two years before childbirth stratified by age group measured as the attributable proportion (AP) of the risk in the groups that are doubly exposed to both intermediate or low educational level and minor or moderate/severe mental health condition, AP [95% CI]

Maternal age, years	Educational level	Mental health condition (concidered two years before childbirth)		
		No	Minor	Moderate/severe
≤23	High/intermediate			
	Low		-0.10 [-0.35;0.14]	0.03 [-0.16;0.22]
24-30	High			
	Intermediate		-0.03 [-0.16;0.10]	0.01 [-0.15;0.16]
	Low		0.13 [-0.01;0.28]	0.14 [0.00;0.29]
≥31	High			
	Intermediate		-0.01 [-0.16;0.13]	0 [-0.17;0.17]
	Low		0.10 [-0.10;0.30]	0.13 [-0.04;0.31]

Supplementary table s21

Supplementary table s21: Attributable proportion (AP) of the risk in the group that is doubly exposed to both age≤23 or ≥31 years and each combination of educational level and mental health conditions concidered two years before childbirth, AP [95% CI]

Educational level	Mental health condition (concidered two years before childbirth)	Maternal age, years		
		24-30	≤23	≥31
High*	No			
	Minor		0.05 [-0.14;0.23]	0.04 [-0.09;0.17]
	Moderate or severe		0.02 [-0.19;0.23]	0.11 [-0.05;0.26]
Intermediate	No			0.10 [0.05;0.15]
	Minor			0.13 [0.01;0.26]
	Moderate or severe			0.16 [0.02;0.30]
Low	No		-0.10 [-0.20;-0.01]	0.21 [0.13;0.29]
	Minor		-0.31 [-0.57;-0.05]	0.19 [-0.01;0.38]
	Moderate or severe		-0.20 [-0.36;-0.03]	0.24 [0.08;0.40]

**For women aged≤23 years high and intermediate educational level was merged*
